# Modelling shifts in social opinion through an application of classical physics

**DOI:** 10.1038/s41598-022-09165-1

**Published:** 2022-03-31

**Authors:** Daniel S. Zachary

**Affiliations:** Advanced Academic Programs, Johns Hopkins University, 1717 Massachusetts Ave., N.W., Washington, DC 20036 USA

**Keywords:** Climate-change policy, Physics, Climate sciences

## Abstract

This paper explores the abstraction of classical physics and applies several metrics that explore the evolution of social opinion. These metrics include an abstraction of Newtonian kinematics: mass, position, speed, acceleration, and Newtonian dynamics, an abstraction of force. Poll data is fit to a 2nd-order polynomial and a logistic function. These fits are used to understand the acceleration of opinion shift, and we explore recent social, cultural, and environmental trends, such as views on global climate change. We compare our results with the evolution of communication technologies and time spent on devices over the past 120 years. We show that the model connects the evolution in opinion with an abstraction of a Galilean concept: acceleration is independent of mass. Finally, we discuss the model of social polarization and the non-linear effect of media such as echo chambers.

## Introduction

Poll taking is a well-established research tool to assess public opinion from a particular sample, for example, recited in this complete compilation of polls taken by Gallup through 2005^1^. Polls reveal social and cultural change and, by their very nature, are averaging data using binary-type questions (e.g., Do you support or don’t support a particular issue?). Details of the make-up of options, the strengths of opinion, the polarity of views are hidden within this averaging. Several papers have explored social change, particularly the shifting of persons within various social roles and social conditions classifications. One such paper used Markov chains to examine temporal changes in frequency distributions within categories defined by social roles and other social conditions^2^. In this approach, the author, Kenneth Land, explores two populations and their social statistics from the earlier work of Sheldon and Moore^3^, where time-series data on changes in the American society (and other societies) have been systematically collected in ever-increasing numbers since the 1950s. In this approach, Land points out the usefulness of the mathematical approach. However, he pointed out that among various shortcomings of the model, perhaps the most looming problem resulted in reducing a complicated system of “estimated structural equations to a few meaningful concepts”^2^. A complementary method includes modeling social interaction using Cellular Automata (e.g., Nowak and Lewenstein^4^), where they studied intrinsic dynamics and emergent properties in populations. Similarly, systems theory provides an academic framework to study complex social phenomena and potential emergent properties. The approach divides complex systems (e.g., society) to smaller components (e.g., individuals)^5^. As individuals interact with each other, emergent properties can appear. In this context, individuals or a group may act differently or unpredictably than if individuals acted alone or even if all known properties of interaction were considered^6^. Other authors explore social systems, such as multi-agent systems, to better understand social structure and norms^7,8^, or the impact of social change and various initiatives^9^. Still, other approaches include studies of social interaction via different media intervention and collective action in role modeling (e.g., Bilali et al.^10^).

The challenge of simulation in modeling a complex system such as human decision, opinion, and behavior is multi-fold^11^. Early on, Abbott points out that significant historical transformations are not amenable to representation through existing simulation models^12^. The number of degrees of freedom in social phenomena is extensive and constantly changing, making even uniform changes (e.g., large-scale changes in segregation) hard to identify the underlying causes and drivers^13^. For example, the role of emotions in decision processes is one of many such complex phenomena that even if a simulation can attribute a good result, the underlying features are difficult to identify^14^.

The above approach falls into a class of micro-level models that address social behavior at the individual level via agent and simulation. An alternative approach is to study social behavior and hence opinion change in a global view using macro-level models. These models provide a view of the collection of underlying mechanisms that drive change. This paper takes this approach and addresses social change regarding the ensemble of human decisions in a large population (millions) over an extended period (at least 10 years). In this vein, the modeling of many interactions resembles physical systems where myriad small interactions produce a net effect.

The influence of mass media has dramatically increased, especially in recent years^15,28^. This recent influence begs the question, how much does it influence our opinions? What are the underlying forces that motivate opinion change? Are the drivers for opinion change individuals who decide on their own after learning about a topic, or is opinion change primarily media-driven and opinion shift occurs because most individuals are influenced by media personalities that convince the listener what they should believe? The answer is likely a combination of these two views. Several authors have done extensive research on this topic^16–18^. The question remains: how much do others influence us?

This paper poses a new approach to understanding social change using an abstraction of Newtonian laws. Though perhaps unorthodox approach, we show that abstraction of physical laws and principles produces dynamics and can explain long-term cultural trends and and opinion shifts. We parallel classical kinematics and dynamics, though we do not focus on individual dynamics, only the ensemble effects. Though quantum mechanics can treat the micro view of physical reality, this tool is generally inappropriate to explore phenomena at larger scales where classical mechanics is appropriate. We use this framework to explore both long-term trends in culture and opinions on environmental goals, namely views towards climate change.

One of the older polls in the USA includes the Gallup Polls, founded by George Gallup in 1935 and continues to provide numerous polls on various social opinions. This poll offers a long baseline of information for this study^19^.

We proceed by (1) formulating a generalization of Newton’s Laws or an *abstraction of Newtonian kinematics* to study abstract motion. In “Methods”, we describe the abstraction of classical physics and present poll data from the Gallup polls. Next, we present (2) a comparison of the model with information extracted from curves that fit the poll data (“Results”). Finally, in “Discussion”, we discuss the results in the context of social models and explore other areas where the abstraction of classical mechanics may help study non-linear social behavior.

## Methods

### Social data and non-linear behavior

**Figure 1:**
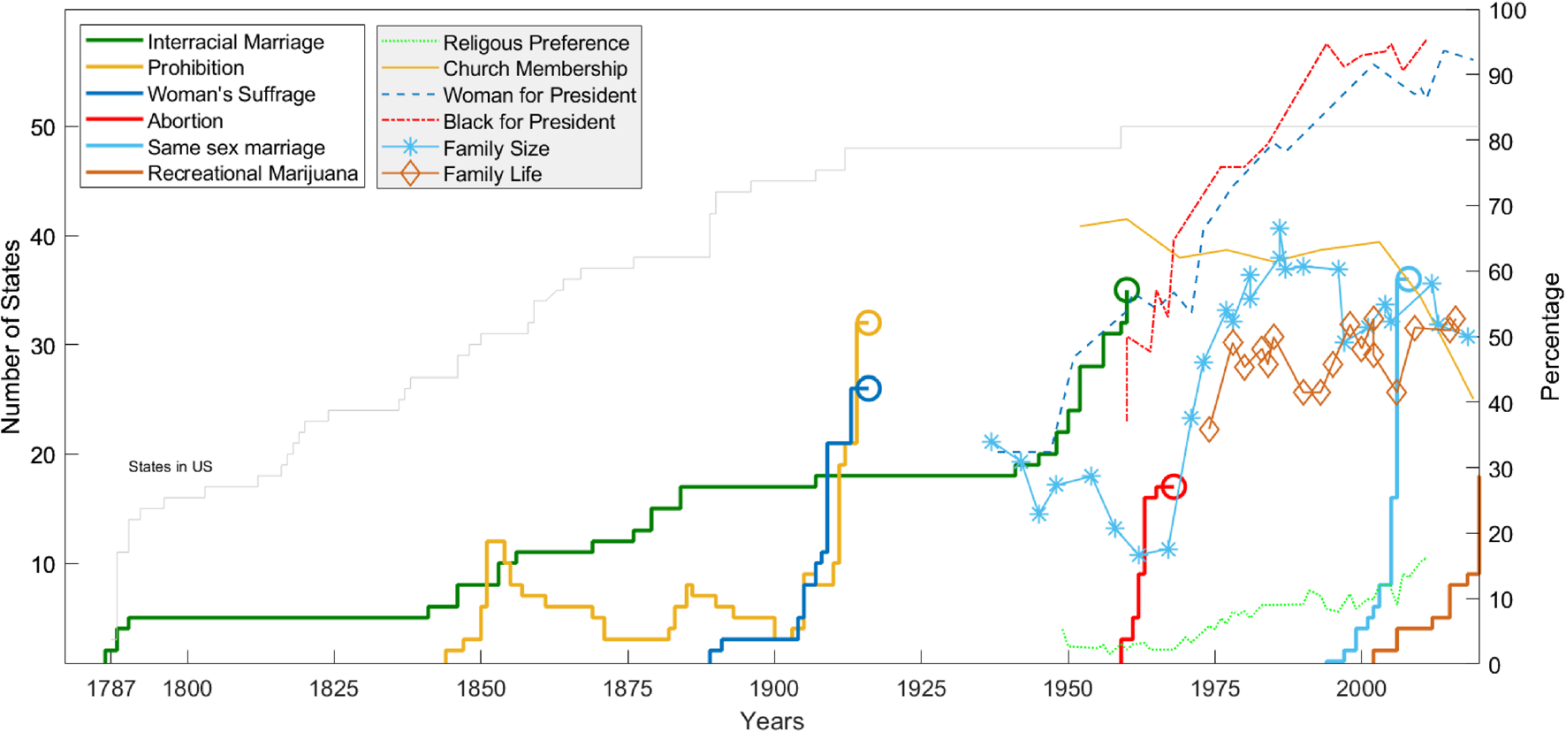
The number of states that support various social issues (thick lines) and Gallup Polls on different trends (thin lines, dots, dashes, and symbols).

To test the *abstraction of Newtonian kinematics* (ANK), we use Gallup polls of long-term trends in social opinion for several significant concerns in the USA^19,20^. Data is shown in Fig. 1. The left vertical axis displays the number of states for the first set (left legend) of data and the right hand axis shows percentage for the second (right legend). We also include PEW Research data on recent polling on environmental and climate data^21^.

The plot indicates the evolution of states on their opinion to either remove a ban or increase a prohibition. We divide the data into two sets of information: (1) data showing states that support (or prohibit) issues and (2) data of polling of popular topics. The former are indicated with thick lines in the figure, and circles at the end of these lines indicate a constitutional amendment or Supreme Court decision. The latter set of data include various trends: percentage of the population with no religious affiliation church membership, willingness to vote for a woman for president, desire to vote for a black president, ideal family size (Family Size), and woman’s preference to work outside of the home (Family Life)^22,23^.

**Figure 2:**
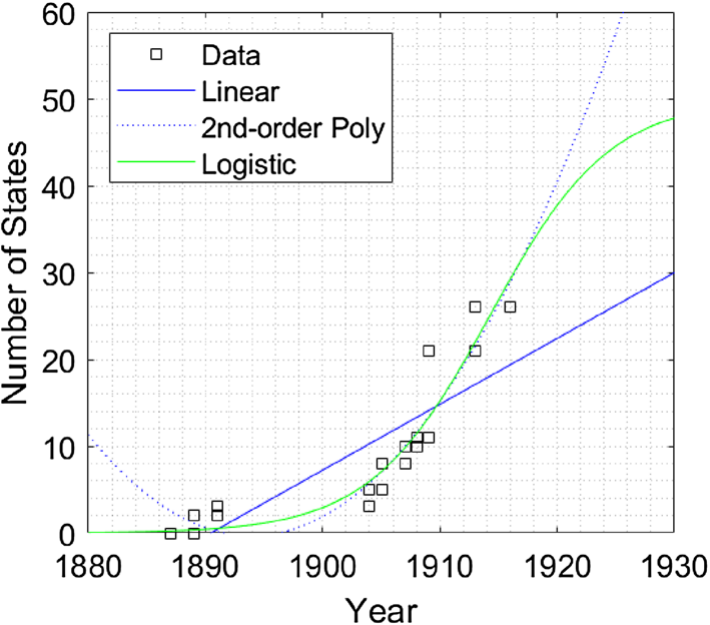
Example of data, Women’s Suffrage (S3).

**Figure 3:**
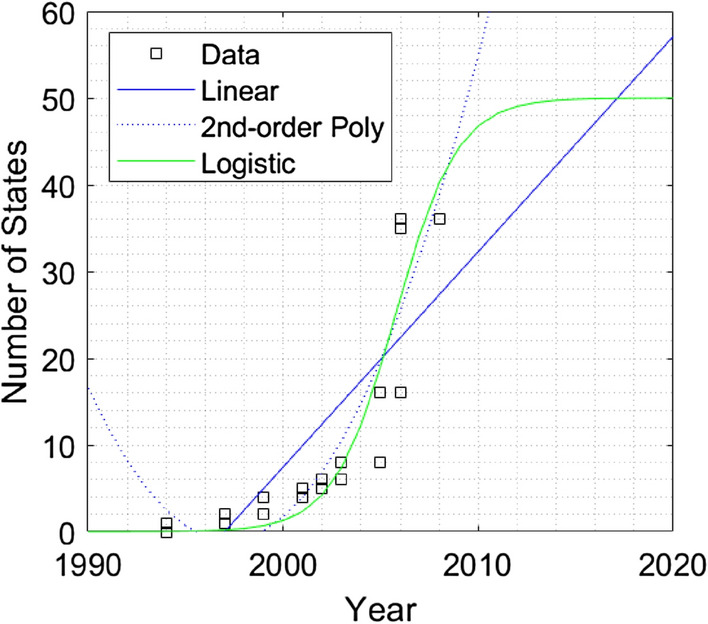
Example of data, same-sex marriage (S5).

Two examples demonstrate the linear, polynomial, and logistic fits to the data, see Figs. 2 and 3. Additional figures showing the complete set of polls, are shown in Figs. 6 and 7. The full set of fits are shown in the Appendix C. We also show the non-linear behavior of each poll by comparing the $R^2$ for linear, 2nd-order polynomial, and logistic fits. A short description of the poll is given in Table 1. A linear fit might initially describe rising (or descending) data from Gallup polls. Yet the trend is generally better fit by a 2nd-order polynomial or logistic curve as opinion starts to change faster. The logistic curve,

1 $$\begin{aligned} P(t)= \frac{a}{1+e^{-k(t-t_o)}}, \end{aligned}$$

describes an evolving phenomenon and the value *a* characterizes a leveling off of the data. The maximum values are $a=100$ for percentage data, or $a = 50$ for the states data. The values *k* and $t_o$ are fit to the data. Appendix B provides additional information on the logistic fit for this data.

### Newton’s laws of motion

We define the abstraction of “distance” or “position” of people’s opinion from a specific position, $x \longrightarrow \widetilde{x}$, the abstraction of mass or the inertia to change of opinion from a norm $m \longrightarrow \widetilde{m}$, the abstraction of the rate of change opinion or speed, $v \longrightarrow \widetilde{v}$, and the abstraction of momentum, the product of the mass and speed, $p \longrightarrow \widetilde{p}$. A natural extension of the abstraction of Newtonian kinematics is Newtonian dynamics (study of force). The abstraction of force, the push, pull or influence “acting” on a population with an opinion, $F \longrightarrow \widetilde{F}$. The notion of time remains the same in the abstraction.

**Table 1:** Gallup polls^19^ showing social positions, environmental and climate data^21^ along with $R^2$ fit values. The best fits are in bold.

Name	Description	$R^2$
		Linear/2nd-ord. poly/logistic
**Tracking change in states**		
Interracial marriage (S1)	Number of states that support interracial marriage, up through the Loving vs. Virginia Supreme Court decision of 1967	0.878/**0.902**/0.896
Prohibition (S2)	The number of dry states prior to the 18th Amendment (Prohibition)	0.268/**0.493**/0.363
Woman’s suffrage (S3)	Number of states that let women vote in presidential elections before the 19th Amendment took effect	0.683/0.892/**0.899**
Abortion (S4)	Number of states that allowed at least some abortions before the Supreme Court ruled in Roe vs. Wade Decision	0.798/**0.829**/0.703
Same-sex marriage (S5)	Number of states that allowed same-sex marriage before the Supreme Court ruled in Obergefell vs. Hodges in 2015	0.711/0.920/**0.928**
Recreational marijuana (S6)	Number of states where recreational marijuana is legal	0.725/0.813/**0.823**
**Polling of popular topics**		
Non-religious affiliation (O1)	Percentage of Americans with no religious affiliation (1948–2009)	0.893/**0.899**/0.890
Church membership (O2)	Trends in American attendance in church/synagogue/mosque (1952–2019)	0.619/**0.754**/–
Women for president (O3)	American’s willingness to vote for a woman for president (1937–2019)	0.935/**0.955**/–
Black for president (O4)	American’s willingness to vote for a black president (1958–2019)	0.904/**0.967**/–
Ideal family size (O5)	Americans’ views on ideal family size with no more than two children in a family (1936–2018)	0.520/**0.568**/0.466
Woman’s work preference (O6)	Woman’s preference in America to work outside of the home (1974–2016)	0.390/**0.391**/0.363
**Environmental and climate topics**		
Protecting the environment (E1)	Percentage of Americans who support prioritizing policies to protect the environment (2009–2020)	0.802/**0.819**/0.802
Stricter environmental laws (E2)	Percentage of Americans who say stricter environmental laws are ’worth the cost’ (1994–2019)	0.802/**0.819**/0.802
Climate change issues (E3)	Percentage of Americans who support prioritizing policies to mitigate against climate change (2009–2020)	0.803/**0.939**/0.836

**Figure 4:**
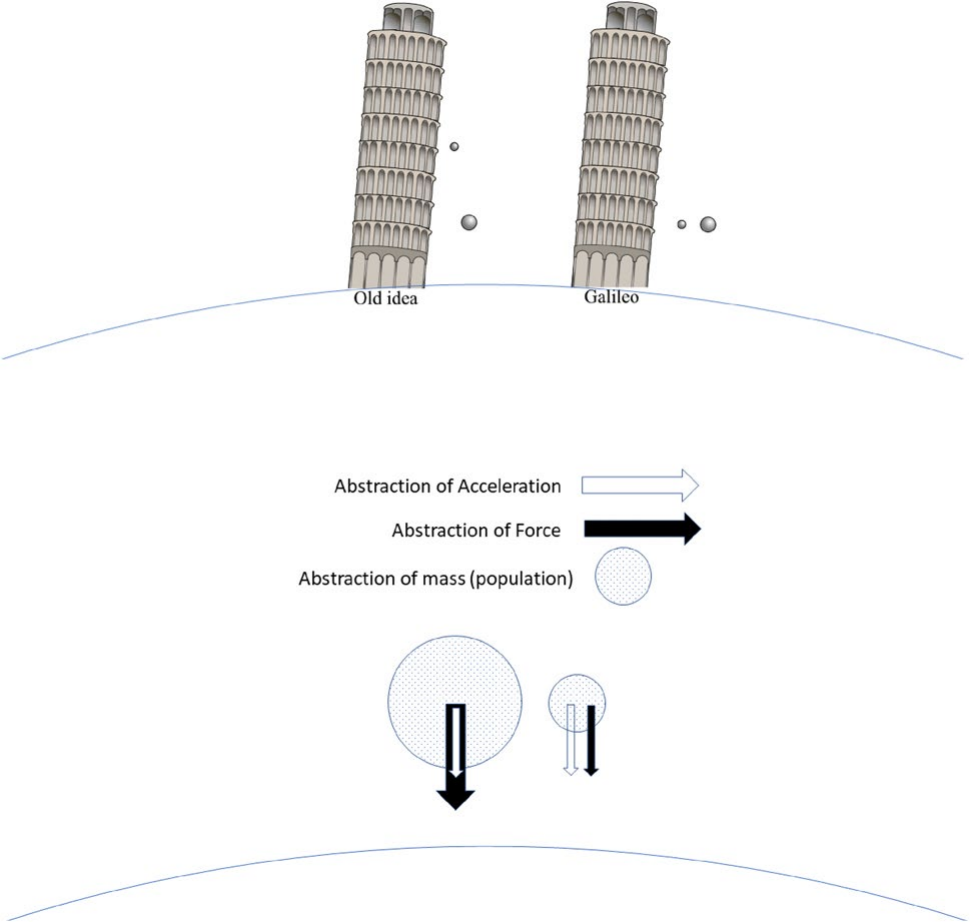
Abstraction of the acceleration and force with reflection to Galileo’s experiment of 1591^24,25^.

Energy can also be considered in the abstraction. Potential energy can invoke the change of opinions $\widetilde{V}$ and kinetic energy $\widetilde{T}$, the energy associated with the motion or change in opinion, towards or away from a norm. The lack of physical dimensions means that the abstraction is meaningful for speed but not a vector quantity such as velocity. The abstraction represents kinematics created from a constant force, not different than the equations of motion generated from the Lagrangian of an object in a (nearly constant) gravitational potential such as the one found near the Earth’s surface. A sketch of the abstraction is given in Fig. 4. Here we see Galileo’s famous experiment reproduced. He ascended to the top of the Tower of Pisa and subsequently dropped a massive and less massive ball. The contemporary thought was that the more massive ball would fall faster. Still, instead, both fell at the same rate, dispelling the centuries-old Aristotelian theory of gravity stating that objects fall at speed proportional to their mass. For completeness, the abstraction of the three classical laws are shown in Table 2. Appendix A discusses the abstraction of Newton’s 3rd law.

**Table 2:** Newton’s Laws and an abstraction.

Law	Description	Equation	Abstraction
1st	Law of Inertia: an object at rest will stay at rest, and an object in motion will continue at a constant speed and in a straight line unless acted on by a net external force	$\sum \mathbf {F} =0\;\Leftrightarrow \;{\frac{\mathrm {d}\mathbf {v}}{\mathrm {d}t}}=0$	The opinion of a population will remain as it is unless a net driving force (influence) acts upon it
2nd	The rate of change of momentum of a body over time is directly proportional to the net driving force and moves in the same direction as the applied force	$\mathbf {F} ={\frac{\mathrm {d} (m\mathbf {v} )}{\mathrm {d} t}} = m\,{\frac{\,\mathrm {d} \mathbf {v} \,}{\mathrm {d} t}} = m\mathbf {a}$	The rate of change of the abstract momentum is directly proportional to the external influence (force) applied and occurs in the same direction as the force
3rd	All forces between two objects exist in equal magnitude and opposite direction. If one object *A* exerts a force $F_A$ on a second object *B*, then *B* exerts a force $F_B$ on *A*, and the two forces are equal in magnitude and opposite in direction	$F_A = -F_B$	All influences acting on a population exists in equal magnitude and opposite direction: if one object A exerts a force $F_A$ on a second object B, then B exerts a force $F_B$ on A, and the two forces are equal in magnitude and opposite in direction

The equations of motion for an object under constant force becomes,

2 $$\begin{aligned} x(t) = x_o + v_o t + a t^2/2, \end{aligned}$$

with the natural abstraction,

3 $$\begin{aligned} {\widetilde{x}} (t) = \widetilde{x}_o + \widetilde{v}_o t + \widetilde{a}t^2/2, \end{aligned}$$

and therefore the acceleration and force becomes,

4 $$\begin{aligned} \widetilde{a}= \frac{2 \cdot (\widetilde{x}(t)-\widetilde{x}_o - \widetilde{v}_o t)}{t^2} = { \frac{\widetilde{F}}{m}}, \end{aligned}$$

demonstrating that the force is proportional to the mass.

### Extracting the data to produce acceleration and force

Acceleration and force are extracted from Fig. 1 using logistic fits for each poll. The time *t*, or year, is marked at the inflection point of the logistic curve. This is shown in Figs. 2 and 3 for years 1915 and 2005 respectively. Acceleration is determined at the inflection point and time *t* is measured from the first poll data point $t_1$ to the inflection point $t_{inf}$, or $t ~=~ t_{inf} - t_1$. Likewise, the position, $\widetilde{x}(t)$ represents the “distance” along the y-axis in Figs. 2 and 3, measured from the first data point to the inflection point, or $\widetilde{x}(t) ~= \widetilde{x}(t_{inf}) - \widetilde{x}(t_1)$ . The initial velocity $\widetilde{v}_o$, is determined using the values of the fitted curve at the first two data points or,

5 $$\begin{aligned} \widetilde{v}_o = \frac{\widetilde{x}(t_2) -\widetilde{x}(t_1)}{t_2 - t_1}. \end{aligned}$$

**Figure 5:**
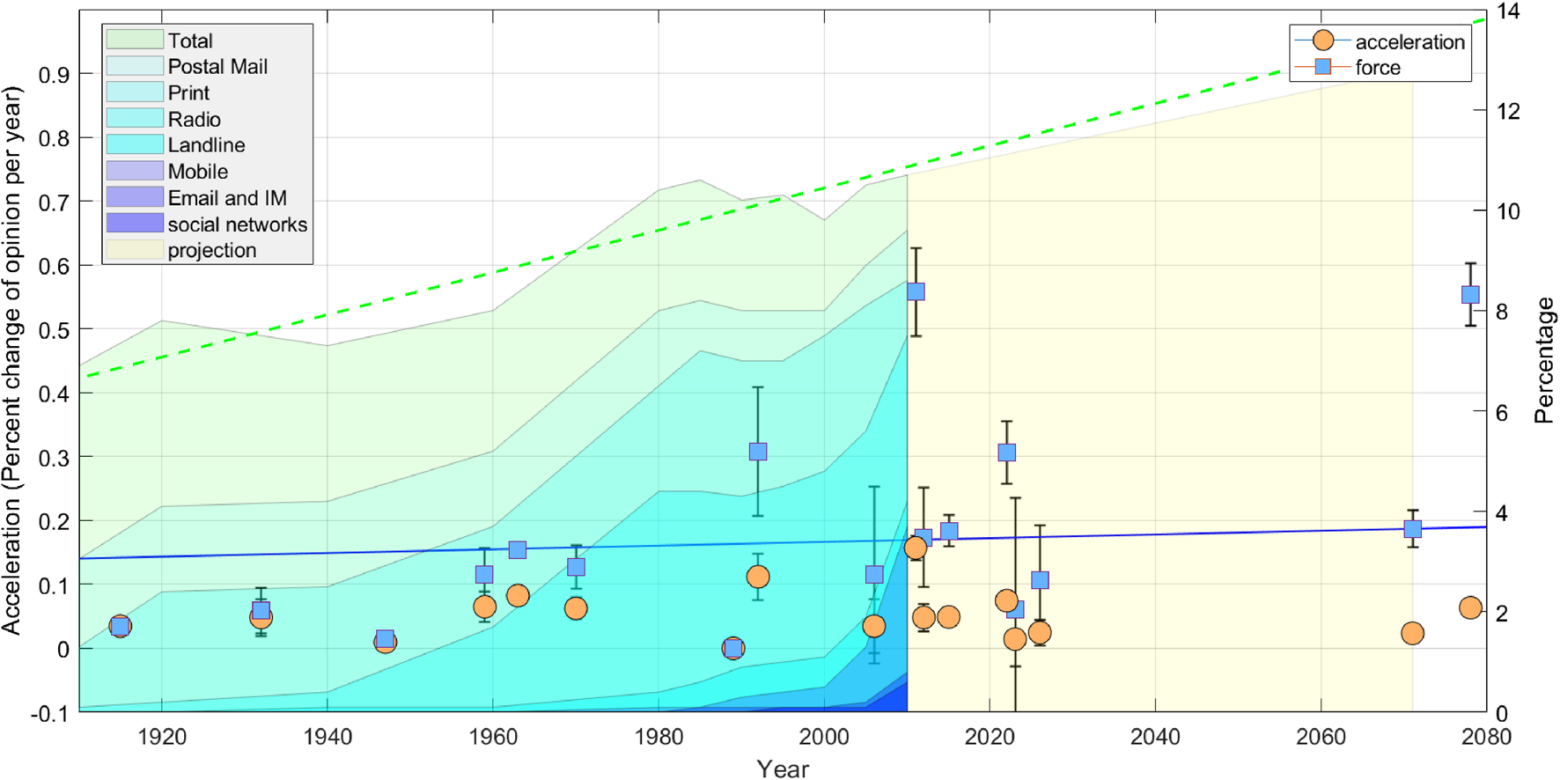
Acceleration and force versus year. Details of the technologies, fits, and data are given in the text.

Table 3 provides the values for *t* (year), $\widetilde{x}_{inf}$ and $\widetilde{v}_{inf}$ at the inflection point for each social and environmental poll. The acceleration $\widetilde{a}$ is determined using Eq. (4) at the inflection points for each poll and plotted verses year in Fig. 5. Likewise, the force term $\widetilde{F}= m\widetilde{a}$ is determined using a (unitless) abstraction of mass, or value normalized to the USA population in 1910, $\widetilde{m}_{1910} = 1$.

**Table 3:** Kinematic values for logistic fits to poll data, including initial time $t_1$, initial velocity, $\widetilde{v}_o$, and values at the inflection point, $t_{inf}$, $\widetilde{x}_{inf}$, and $\widetilde{v}_{inf}$.

	S1	S2	S3	S4	S5	S6	O1	O2	O3	O4	O5	O6	E1	E2	E3
$t_1$	1786	1844	1887	1959	1994	2002	1949	1952	1938	1960	1937	1974	2009	1994	2009
$\widetilde{v}_o$	0.06	0.01	0.0	0.0	0.0	0.0	0.15	− 0.25	0.93	1.6	0.46	0.17	1.95	− 0.13	2.20
$t_{inf}$	1947	1932	1915	1970	2006	2026	2071	2023	1959	1963	1989	2011	2015	2078	2022
$\widetilde{x}_{inf}$	25	25	25	25	25	25	50	50	50	50	50	50	50	50	50
$\widetilde{v}_{inf}$	0.20	0.49	2.44	2.91	7.79	2.07	0.74	− 0.29	1.05	1.78	0.51	0.19	2.01	− 0.14	2.58

The errors for each fit are determined using the 95% confidence interval determined using the standard MATLAB fit routine (MathWorks$\circledR$) and constraining the curve to a maximum of either 50 or 100%. This error is subsequently used to determined the uncertainty in $t_{inf}$. Uncertainties for $\widetilde{x}$, and $\widetilde{v}_o$ are determined from statistics in the Gallop polls, ranging from 1000 to 4000 for each data point. Finally, standard error propagation rules are applied to Eq. (4) to determine the error for the acceleration and the normalized force.

## Results

The evolution of acceleration (circles) and normalized force (squares) are plotted in Fig. 5 along with their respective uncertainty for each poll data. One data outlier is seen in Fig. 5, the 2006 data point (Same-Sex Marriage). This point is characterized by a short base (short time between the first and last poll data) producing a large fit error. Two additional opinion polls were considered and plotted. These two points complement S1 and S6, polling for interracial marriage (inflection point at 1992) and the legalization of marijuana (inflection point at 2026). The 2078 point, the most projected point, represents polls for stricter environmental laws (E2) and results from data fitting to the slow rise of the logistic curve, producing an inflection point projected decades into the future. Similarly, the non-religious point (O1) is fitted to polling data that is slow rising, also producing an inflection point far off into the future.

Within statistical error, the evolution of the acceleration for the three data sets (Table 1) is flat, $3 \times 10^{-5} \pm 0.0005$ percentage points per year. Three environmental polls (data at the bottom of Table 1) have been included in the analysis. The normalized force increases by $0.002 \pm 0.0025$ percentage points per year (blue, solid line), or also flat within error. The increase, though larger than the acceleration, is nonetheless statistically insignificant. Technologies include (light green to dark blue or top to bottom in the figure) total media, postal mail, printed paper, radio, landline telephones, mobile phones, emails, and other internet messaging (including text and instant messengers) social networks. The projection of the total is also provided through 2070 (in yellow) The force increases similarly to the time spent on using (or reading) the above technologies.

In contrast, the time spent listening to the radio, interacting on social networks, and accumulating time for all technologies rises faster than the force. The slope of the accumulated time on media is $0.042 \pm 0.011$ percentage points per year (green, dash line). For comparison, the yellow trapezoid in the figure represents an extension of the current technology trend , measured from 2005 to 2010 and extended to 2071.

## Discussion

The time spent on this new media format may provide users with a critical level of selective reinforcement. However, this change did not increase in acceleration. Though a slightly larger increase is seen in the force term, it is also statistically insignificant (Fig. 5). This abstraction of Newtonian mechanics is used to study the rate of opinion change of several social and environmental topics. The main finding is the rate of change of opinion has remained flat over the past 120 years. Returning to the physical analogy, a force provides acceleration on an object, the equivalent of a second-order derivative with respect to time, or *rate of change of position with respect to time*. This same dynamics is found in the *rate of change of opinion with respect to time*. The zero acceleration change might indicate that social media does not play a role in the changing opinion as a whole. On the other hand, micro-processes, including polarization of opinion, would be averaged out and therefore remain undetected in this analysis.

### Areas to explore

The polarization of opinion can result from drivers, including media storms^29^, feedback loops (media affecting personalities and personalities affecting media attention), and information “echo chambers”^30–32^. Fits using a 2nd-order polynomial or the logistic function characterize the average acceleration of evolving opinion, but two polarized opinion subsets would remain hidden in our analysis. Understanding these non-linear effects is critical for better understanding the dynamics of polls and better discerning the malicious spread of misinformation^30^. Polarization might be studied in a similar vein as we approached this research using classical mechanics, but now using an approach for many-body interactions, the framework of thermodynamics. This approach might help elucidate underlying mechanisms and complement recent social simulations exploring this issue^33^. A further interest includes pursuing how media influences polarization; in theory, we could similarly address polarization, using the methods of this, yet exploring smaller subsets of the population that is influence to various degrees by echo chamber phenomena and other media effects.

## Conclusions

Polls measuring the change in opinion have been analyzed using an abstraction of acceleration. We show that acceleration is flat over 120 years data. On the other hand, when the population is considered, the abstraction of force rises slightly over time, yet it too, is statistically insignificant. The effects of media, including social media, do not seem to alter the acceleration of opinion as demonstrated over one hundred years of poll taking. The study explores averages and the population may experience more rapid changes in opinion over time, but these are averaged out when considering the opposing opinions.

## Supplementary Information

Supplementary Information.

## Data Availability

All data used in this study are available from the corresponding author on request.

## References

[1] The Gallup Poll, Public Opinion 2005, Rowman & Littlefield (eds. Gallup, A., Gallup, A. M., Newport, F.) (Gallup Organization, 2006).

[2] Land Kenneth, C. *Modeling Macro Social Change, Sociological Methodology*, vol. 11 219–278 (Wiley, 1980) (**direct quote from p. 271**).

[3] Sheldon EB, Moore WE (1968). Indicators of Social Change: Concepts and Measurements.

[4] Nowak A, Lewenstein M, Hegselmann R, Mueller U, Troitzsch K.G (1996). Modeling social change with cellular automata. Modelling and Simulation in the Social Sciences from the Philosophy of Science Point of View. Theory and Decision Library (Series A: Philosophy and Methodology of the Social Sciences).

[5] Waldrop M. Mitchell (1992). Complexity: The emerging science at the edge of order and chaos.

[6] Conte, R. *et al*. Emergent and immergent effects in complex social systems. In *AAAI Fall Symposium: Emergent Agents and Socialities* (2007).

[7] Hexmoor H, Venkata SG, Hayes D (2006). Modelling social norms in multiagent systems. J. Exp. Theor. Artif. Intell..

[8] Uhrmacher, A. M., & Weyns, D. (eds.) *Multi-Agent Systems: Simulation and Applications*, 1st ed. (CRC Press, 2009).

[9] Hirsch GB, Levine R, Lin Miller RL (2007). Am. J. Community Psychol..

[10] Bilali R, Vollhardt JR, Rarick J, David R (2017). Modeling collective action through media to promote social change and positive intergroup relations in violent conflicts. J. Exp. Soc. Psychol..

[11] Garson GD (2009). Computerized simulation in the social sciences: A survey and evaluation. Simul. Gaming.

[12] Abbot A (1988). Transcending general linear reality. Sociol. Theory.

[13] Abbott A (2001). Time Matters: On Theory and Method.

[14] Lane, R. D. & Nadel, L. (eds) *Cognitive Neuroscience of Emotion* (Oxford University Press, 2000).

[15] Tichenor PA, Donohue GA, Olien CN (1970). Mass media flow and differential growth in knowledge. Public Opin. Q..

[16] Scheufele D, Tewksbury D (2007). Framing, agenda setting, and priming: The evolution of three media effects models. J. Commun..

[17] Strömberg, D. Media coverage and political accountability: Theory and evidence. In *Handbook of Media Economics*, vol. 1 (eds. Anderson, S., Strömberg, D., & Waldfogel, J.) (2015).

[18] Sema, P. Does social media affect consumer decision-making? *MBA Student Scholarship*. **24** (2013). https://scholarsarchive.jwu.edu/mba_student/24. Accessed on August 2021.

[19] Gallup Global Research. https://www.gallup.com/analytics (accessed 19 July 2021).

[20] New, J. Visualizing the Pace of Social Change, Center for Data Innovation, credit to Alex Tribou and Keith Collins of Bloomberg Business https://datainnovation.org/2015/04/visualizing-the-pace-of-social-change/ (2015). Accessed on August 2021.

[21] Funk, C., & Kennedy, B. How Americans see climate change and the environment in 7 charts, originally. In *As Economic Concerns Recede, Environmental Protection Rises on the Public’s Policy Agenda* (Accessed 21 April 2020).

[22] Saad, L. 10 Major Social Changes in the 50 Years Since Woodstock. (Accessed 22 July 2021) https://news.gallup.com/opinion, Gallup Blog (2019).

[23] Newport, F. In *U.S., Increasing Number Have No Religious Identity Modest Increase Since 1990s in Percentage Who Believe Religion is Out of Date* (2010).

[24] Knott, T. Sketch of leaning tower from Theresa Knott, uploaded to English Wikibooks (2005).

[25] Hilliam, R., & Galilei, G. Father of Modern Science. In *Rachel Hilliam gives 1591 as the Date Where Galileo Performed this Famous Experiment* 101 (The Rosen Publishing Group, 2005).

[26] Verhulst, P.-F. Recherches mathématiques sur la loi d’accroissement de la population [Mathematical Researches into the Law of Population Growth Increase]. *Nouveaux Mémoires de l’Académie Royale des Sciences et Belles-Lettres de Bruxelles*. **18**, 8 (1845) (**Origin of the logistic curve, retrieved 18 August 2021**).

[27] Malthus, T. R. *An Essay on the Principle of Population as it Affects the Future Improvement of Society, with Remarks on the Speculations of Mr. Goodwin, M. Condorcet and Other Writers*, 1 ed. (J. Johnson in St Paul’s Church-yard, 1798).

[28] Valkenburheueg PM, Peter J, Walther JB (2016). Media effects: Theory and research. Annu. Rev. Psychol..

[29] Walgrave S, Boydstun S, Vliegenthart R, Hardy A (2017). The nonlinear effect of information on political attention: Media storms and U.S. congressional hearings. Polit. Commun..

[30] Del Vacario M, Bessi A, Zollo F, Petroni F, Scala A, CAldarelli G, Stanley H.E, Quattrociocchi W (2016). Quattrociocchi. Proc. Natl. Acad. Sci. U.S.A..

[31] Garrett RK (2009). J. Comput. Mediat. Commun..

[32] Garimella, K., De Francisci Morales, G., Gionis, A., & Mathioudakis, M. In *Proceedings of the 2018 World Wide Web Conference, WWW ’18 (International World Wide Web Conferences Steering Committee, Geneva, Switzerland* 913–922 (2018).

[33] Baumann F, Lorenz-Spreen P, Sokolov Ig. M, Starnini M (2020). Modeling echo chambers and polarization dynamics in social networks. Phys. Rev. Lett..

