# Supplementary material for: Modelling shifts in social opinion through an application of classical physics
Source: Sci Rep. 2022 Mar 31;12:5485. doi: 10.1038/s41598-022-09165-1 (PMC8971447; doi:10.1038/s41598-022-09165-1)
Supplement: Supplementary file 1 — Supplementary Information. [file 41598_2022_9165_MOESM1_ESM.pdf]

Supplementary Material

Survey Questions

This section includes the questions from the surveys used in Figure 1. The questions for the trends of popular topics include[20]:

1. None-religious Affiliation: How important would you say religion is in your own life - very important, fairly important, or not very important?
2. Church Membership: Do you happen to be a member of a church or synagogue?
3. Women for President: If your party nominated a generally well-qualified person for president who happened to be a woman, would you vote for that person?
4. Black for President: If your party nominated a generally well-qualified person for president who happened to be black, would you vote for that person?
5. Ideal Family Size: What do you think is the ideal number of children for a family to have? $0, 1, 2, \ldots, 6$ or more, no opinion.
6. Woman's Work Preference: If you were free to do either, would you prefer to have a job outside the home, or would you prefer to stay at home and take care of the house and family?

Survey questions for environmental and climate topics include:

1. Protecting the Environments: Should protecting the environment be a top priority, important but lower priority, not too important, or should it not be done?[38]
2. Stricter Environmental Laws: Which comes closer to your view — even if neither is exactly right. Stricter environmental laws and regulations cost too many jobs and hurt the economy or stricter environmental laws and regulations are worth the cost?[37]
3. Climate Change Issues: Should dealing with global climate change be a top priority, important but lower priority, not too important, or should it not be done?[38]

Newtonian dynamics and the 3rd law

Though we do not address the abstraction of Newton's 3rd law directly in this paper, apart from the definition in Section 1.2, we do note its relevance. Newton's 3rd law states that a force acting on an object will be accompanied by an equal and opposite force acting on it. For example, a finger pushing a matchbox horizontally and leftward on a table is met by an equal and opposite force, resulting from friction between the matchbox and the table, directed to the right pushing against the finger. In addition to these equal and opposite forces is the additional force (from the arm muscles) that will accelerate the matchbox.

Likewise, an abstraction can be made of the 3rd law to the context of this paper. We argue there is a "force" that "motivates opinion change." This force is met by an equal and opposite resistance we identify as reflection, discussion, disagreement. These resistance forces do exactly that, resist the unhindered "acceleration" of opinion change, creating an equal and opposite force resisting that change. If the motivation is large enough, an "acceleration" or movement in the polls will result. Just as a matchbox accelerates to the left in this example, so will opinion change be reflected as an "acceleration" of change in opinion polls.

Logistic Function and polling data

The logistic function, $f(x) = a/(1+e^{bx})$, or S-curve naturally describes the asymptotic population growth [27] with origins in ecology modeling[28] that models exponential growth with a carrying capacity limitation. The model characterizes a process that grows slowly, accelerates in the growth, and then tapers off to an asymptotic level. In this work, the process (opinion poll data) grows theoretically from 0% in agreement or 0 states in agreement to 100% agreement and 50 states agreeing. Assuming that the opinion polls complete their theoretical trend, the logistic behavior of the polls can be interpreted as falling grains of sands from a bucket. At first, a few grains fall (few people changing their opinion), then many, then few as the bucket is emptied. Saturation occurs as most reach an opinion. The inflection point, marks where the majority of people polled have changed from one opinion to the other. This is an idealized view, of course. Often, poll data plateaus at a level less than the theoretical maximum, or reverse and descend in value, and even fluctuate. Fluctuations, for example are observed in woman's preference to work outside of the home (see Figure 1 and the Family Life data).

Additional figures

This appendix provides the complete set of data, including linear, 2nd order polynomial, and logistic fits, complementing the data shown in Figures 3 and 4.

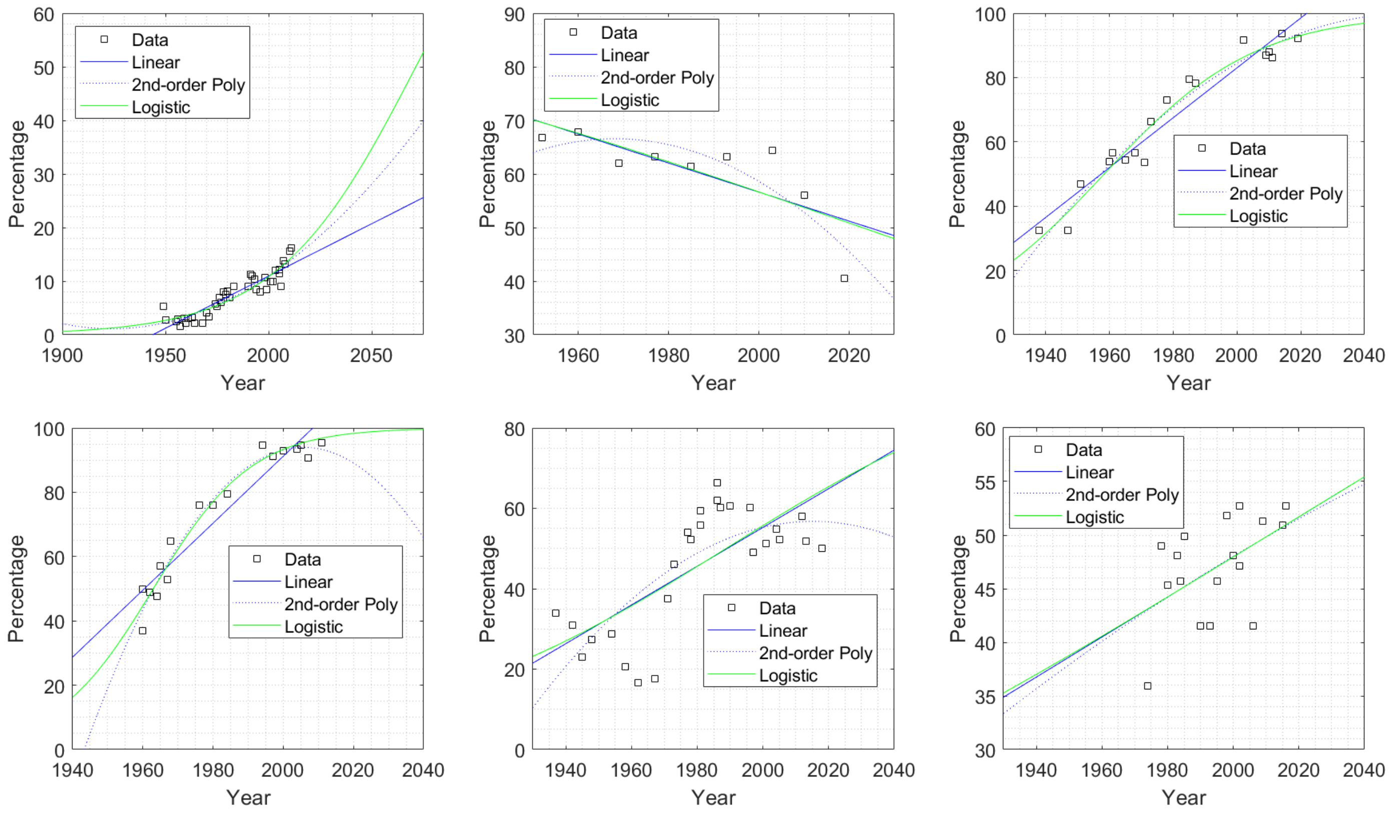

Figure 6. Supplementary figures showing the fits for data, from top left to bottom right: Non-religious affiliation (O1), and Church Membership (O2), Woman for President (O3), Black for President (O4), Ideal Family Size (O5), Woman's Life Style or Work Preference (O6),

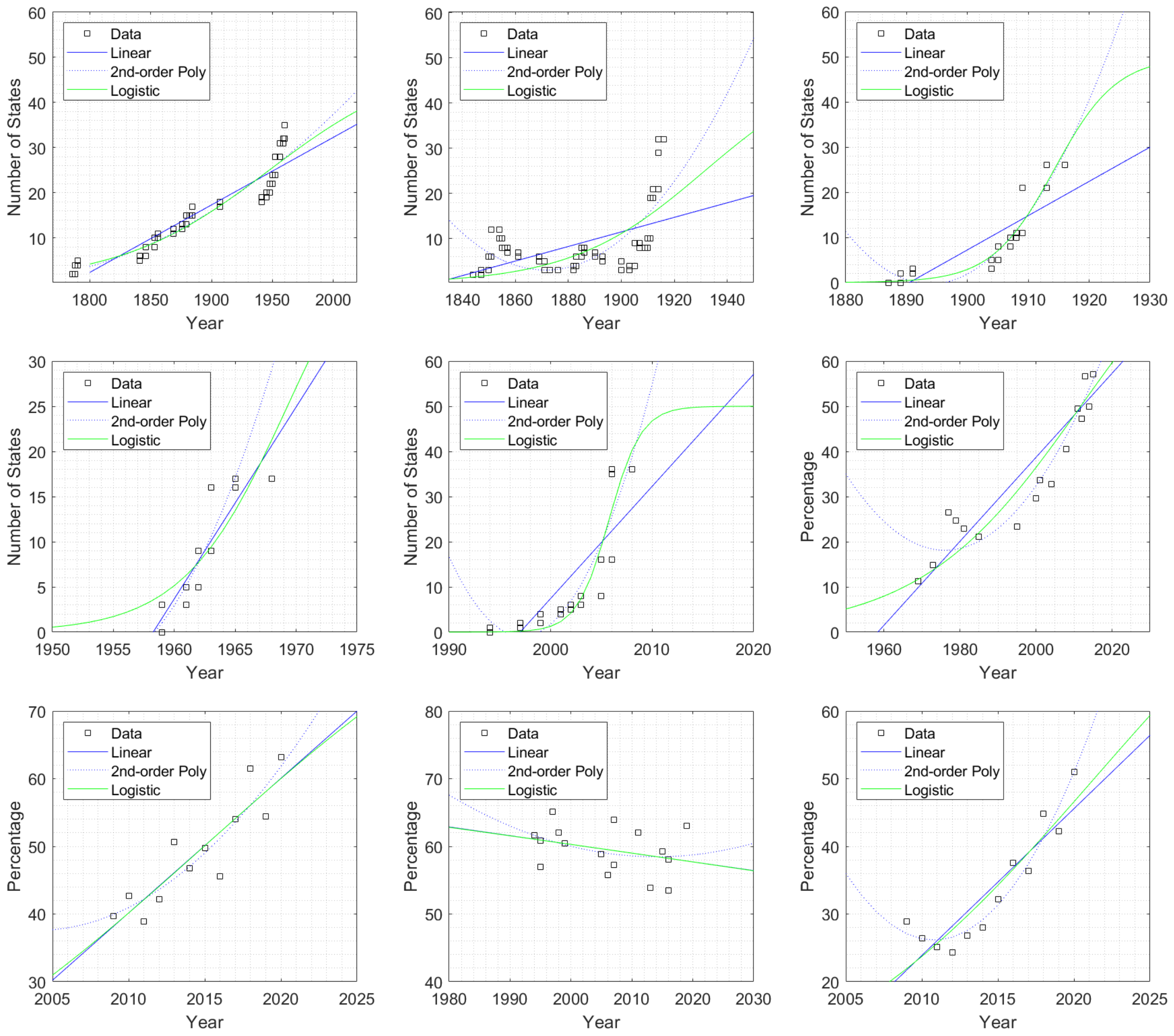

Figure 7. Supplementary figures showing fits for data (continued), from top left to bottom right: Interracial Marriage (S1), Prohibition (S2), Woman's Suffrage (S3), Abortion (S4), Same-Sex Marriage (S5), General Support for Marijuana (S6), Protecting the Environment (E1), Stricter Environmental Laws (E2), and Climate Change Issues (E3).
